# Factors influencing, and associated with, physical activity patterns in dogs with osteoarthritis-associated pain

**DOI:** 10.3389/fvets.2025.1503009

**Published:** 2025-03-19

**Authors:** Christina Stevens, Elizabeth Kawecki-Wright, Avery Rowlison de Ortiz, Andrea Thomson, Savannah Aker, Erin Perry, Emily Haupt, Alejandra Mondino, Masataka Enomoto, Margaret E. Gruen, B. Duncan X. Lascelles

**Affiliations:** ^1^Translational Research in Pain, Department of Clinical Sciences, College of Veterinary Medicine, North Carolina State University, Raleigh, NC, United States; ^2^Department of Clinical Sciences, College of Veterinary Medicine, North Carolina State University, Raleigh, NC, United States; ^3^Comparative Behavioral Research and Thinking Pets Program, Department of Clinical Sciences, College of Veterinary Medicine, North Carolina State University, Raleigh, NC, United States; ^4^Comparative Pain Research and Education Centre, Department of Clinical Sciences, College of Veterinary Medicine, North Carolina State University, Raleigh, NC, United States; ^5^Center for Translational Pain Research, Department of Anesthesiology, Duke University, Durham, NC, United States; ^6^Thurston Arthritis Center, UNC, Chapel Hill, NC, United States

**Keywords:** canine osteoarthritis, canine pain, functional linear modeling, Actigraph, accelerometer, Liverpool Osteoarthritis in Dogs (LOAD), Canine Brief Pain Inventory (CBPI), Clinical Metrology Instruments (CMI)

## Abstract

**Background:**

Accelerometry can be used to measure physical activity and is a validated objective measure for evaluating the impact of osteoarthritis (OA) pain in companion animals. However, several factors other than OA pain can affect physical activity in dogs, and relatively little is understood about their influence. Functional linear modeling (FLM) is an approach for analyzing and visualizing high-frequency longitudinal data such as physical activity and can be used to assess the influence of factors on activity patterns. This study aimed to use FLM to investigate the effect of various factors on physical activity patterns in a cohort of dogs with OA pain.

**Methods:**

Ninety-nine client-owned dogs with radiographic and clinical evidence of OA were fitted with a collar-based activity monitor (Actigraph GT3X). Average vector magnitudes were recorded once per minute over 7 days and averaged to create 24-h, per-minute activity profiles for each dog. Demographic information, owner completed OA Clinical Metrology Instruments (Liverpool Osteoarthritis in Dogs and Canine Brief Pain Inventory), and veterinary examination findings (joint pain, muscle atrophy) were collected. Data were analyzed using FLM and a custom R package to evaluate the effect of each factor on 24-h patterns of physical activity.

**Results:**

At times of peak activity within a 24-h period, dogs with hindlimb OA pain, higher age, higher Clinical Metrology Instrument scores, higher joint pain, greater Body Condition Score and greater muscle atrophy all had decreased activity profiles. However, only age, hindlimb joint pain, and hindlimb muscle atrophy had statistically significant effects on physical activity.

**Conclusions and clinical relevance:**

Several factors influence activity patterns in dogs with OA pain. Understanding what and how factors influence patterns in dogs with OA pain will help refine the usage of physical activity as an objective outcome measure in clinical pain studies.

## 1 Introduction

Osteoarthritis (OA) is a common joint disorder in both companion animals and humans characterized by a deterioration of articular tissues that can be associated with pain (1–3). While OA as a disease of joints can exist without pain, the manifestation of clinical OA (OA-associated pain) is of significant interest due to adverse effects on mobility, function, sleep, social interactions, quality of life, cognitive function, mood, and affect (4–12).

Previous studies have suggested that OA and associated pain is prevalent in 20% of the general dog population (1). However, a more recent report found approximately 37% of dogs presenting to US general practices had a presumptive diagnosis of clinical OA (9). Such estimates are supported by a recent comprehensive evaluation of dogs under 4 years of age where approximately 40% had radiographic evidence of OA. Of these dogs, 60% were identified with at least mild clinical pain on examination, supporting the conclusion that OA pain is highly prevalent in the general canine population (13).

To manage pain associated with clinical OA, methods to detect and measure the impact of pain and the efficacy of treatments are needed (11, 14). Owner-completed Clinical Metrology Instruments (CMIs) (also referred to as Client-Reported Outcome Measures, CROMs) such as the Liverpool Osteoarthritis in Dogs (LOAD) and Canine Brief Pain Inventory (CBPI) have previously been validated to evaluate the impact of OA pain in dogs (15, 16). However, one of the most easily observed signs of clinical OA is a decrease in physical activity (4, 17, 18). Accelerometers have been used to capture these data, but most traditional methods of evaluation have “bucketed” activity or used high level summary statistics with linear mixed-effects modeling rather than evaluating activity profiles globally (2, 5, 19, 20). This traditional approach can obscure differences between groups as subtle details contained within the large volume of longitudinal temporal data may be lost in a bucketed approach (21). Gruen et al. (22) previously identified significant differences within physical activity patterns in a cohort of dogs receiving a therapeutic analgesic, particularly during nighttime and early morning. This relationship, which was not uncovered using traditional analysis, demonstrates the importance of identifying and understanding activity patterns contained within accelerometry data.

Functional linear modeling (FLM) is a computational approach for analyzing and visualizing high-frequency longitudinal data such as physical activity and can be used to assess the influence of factors on activity patterns (21–25). FLM corrects for weaknesses present in traditional modeling by allowing data to be “smoothed” to detect changes in the pattern across a 24-h period (21). The clinical utility of FLM analysis is developing, but has displayed significant potential for evaluating variations in data and the effect of therapeutic agents (22).

Chronic pain conditions that naturally occur in companion animals may offer a more accurate representation of the complex interplay between genetic, environmental, and physiological factors seen in humans than traditional laboratory models (26). The use of companion animals to investigate natural chronic pain conditions, such as OA, holds significant translational potential for testing novel therapeutics aimed at human treatment (27, 28). This necessitates a comprehensive understanding of outcome measures and the factors that influence them.

Therefore, our objective was to investigate the influence of demographic parameters and various measures of function and impairment on activity patterns in a population of dogs with OA pain. We hypothesized that increasing age, body weight, joint pain scores, and owner pain and function assessment scores (CBPI and total LOAD) would lead to decreased activity throughout the day as measured by accelerometry in dogs with OA pain.

## 2 Materials and methods

### 2.1 Patient recruitment and study data overview

Client-owned dogs were recruited between June 2021 and March 2023 for a prospective clinical study of a putative therapeutic for OA pain in dogs. Recruitment was conducted by the Translational Research in Pain (TRiP) Program at North Carolina State University College of Veterinary Medicine (NCSU-CVM), with assistance from the Clinical Studies Core. Methods for recruitment included direct-to-owner (targeted Facebook advertisements, National Public Radio and newspaper advertisements) and outreach to local practices (emails, lunch-and-learn events).

**Figure 1:**
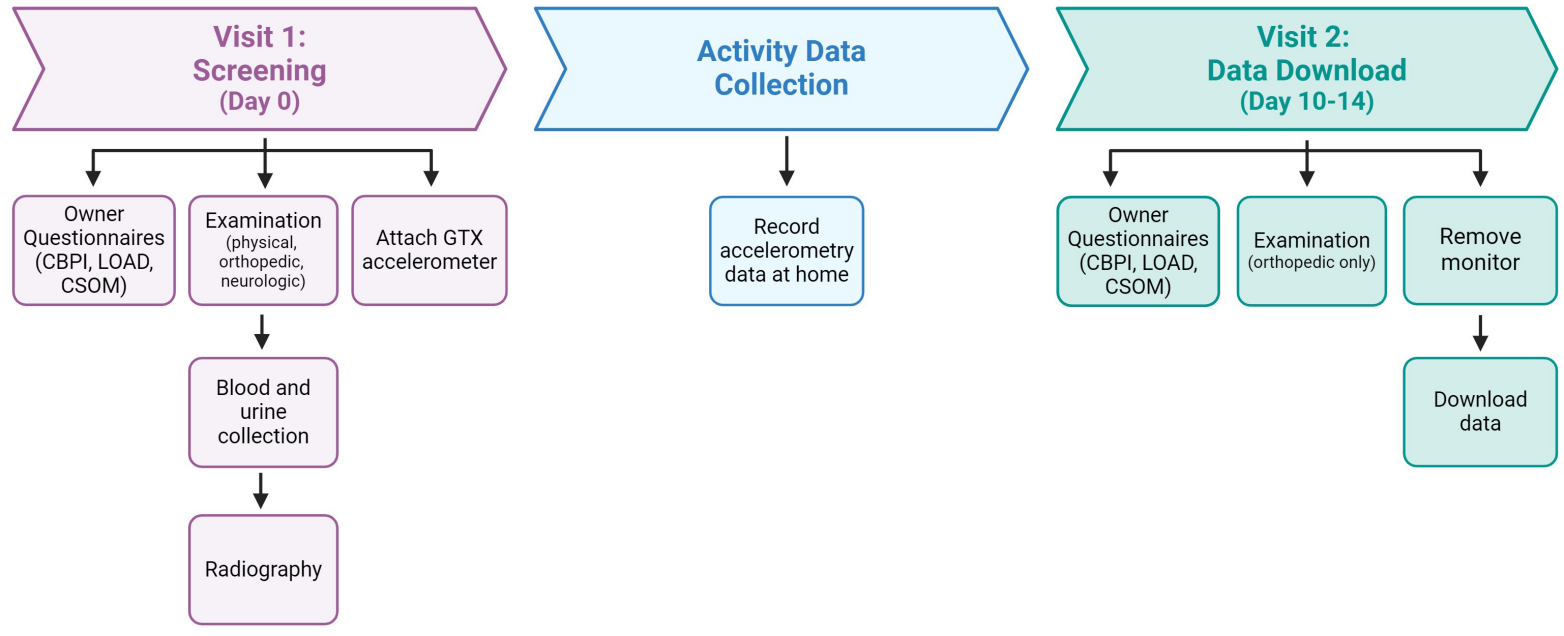
Description of study timeline and activities. LOAD, Liverpool in Osteoarthritis in Dogs questionnaire; CBPI, Canine Brief Pain Inventory questionnaire; CSOM, Client Specific Outcome Measures.

All study-related activities were reviewed and approved by the NCSU Institutional Animal Care and Use Committee (#20-508). All study procedures were explained to owners [first on a pre-screening phone call, and in person at the screening (Visit 1), described below] who provided informed written consent prior to participation. This report used baseline data collected at screening and over the subsequent 10–14 days, prior to randomization to the placebo/investigative therapeutic (which occurred at Visit 2) (Figure 1). This report does not communicate the results of testing the therapeutic for efficacy. All methods described are reported in compliance with the ARRIVE and PetSORT guidelines (29, 30).

All visits and diagnostics were fully funded by the clinical trial. This included radiographs, orthopedic exams, clinical laboratory diagnostics, and any additional diagnostics as deemed appropriate by the study veterinarians. The study assumed financial responsibility for diagnosis and treatment of complications that were determined to be directly and proximately related to the sedation and (although beyond the scope of the current work), complications associated with the investigative product. If the dog successfully completed the 4-month study timeline (regardless of therapeutic intervention), they received a $130 gift card for study completion. No other incentives were provided to encourage participation.

### 2.2 Inclusion/exclusion criteria

Sample size was based on calculations to provide 90% power to detect a difference in mean total LOAD score between the placebo and investigative patient groups of the parent clinical study, and a total sample population of 102 dogs was recruited. Eligible dogs of any breed and sex, at least 6-months of age and weighing at least 7.5 kg were included for evaluation if they had radiographic and clinical evidence of OA (described below). Blood and urine samples were collected to determine eligibility for inclusion into the clinical trial (see below for criteria). Dogs who had been on analgesics (non-steroidal anti-inflammatory medications, corticosteroids or acetaminophen) for OA pain were required to have a 2-week washout interval prior to screening. Adjunctive analgesics (e.g., gabapentin, amantadine) or nutritional supplements were allowed if they were started more than one-month or more than 6-weeks, respectively, before screening and the dog still met the criteria for inclusion with respect to clinical OA. Dogs were required to be in good health as assessed by the clinical veterinarian and was determined by medical history, physical examination, and clinical pathology diagnostic data. Clinical laboratory values for individuals within the study population are provided in Supplementary File 1.

Exclusion criteria included evidence of unstable systemic disease (such as cardiovascular disease with clinical signs, hepatic, renal, or other serious co-existing endocrine conditions), abnormal laboratory results (excepting historically stable, chronic conditions) that could interfere with assessment of efficacy or completion of the study, neurological abnormalities or non-OA disease that affected gait or mobility, fractious behavior at the veterinary clinic, and excessive Body Condition Score (BCS) (9 on a scale of 1 to 9). Dogs who had evidence of acute or unstable orthopedic disease that would benefit from immediate surgical intervention were not included in the study. Dogs who had major surgery within the previous 30-days were excluded. Pregnant or lactating dogs were also excluded.

### 2.3 Screening visit (Visit 1)

#### 2.3.1 Veterinarian assessments

All dogs had a comprehensive assessment performed by a veterinarian (general physical, orthopedic, and neurologic examinations). Assessments were performed within appropriate examination spaces within the NCSU-CVM and conducted by TRiP lab veterinarians (BL, ME, CS, and AO) who received additional training in assessing OA-related pain in companion animal species. Additional recorded variables included age, sex, weight, BCS, and breed.

The examination forms used in this study are provided in Supplementary material and on the TRiP website (https://cvm.ncsu.edu/research/labs/clinical-sciences/comparative-pain-research/clinical-metrology-instruments/ and Supplementary File 2). Orthopedic pain assessment included evaluation of each appendicular joint (forelimb: mani, carpi, elbows, shoulders; hindlimb: pes, tarsi, stifles, hips) and each part of the axial skeleton (cervical, thoracic, thoracolumbar junction, lumbar, and lumbosacral junction) for pain. Each area was evaluated on a five-point scale (0–4) as follows: 0: does not notice manipulation; 1: orients to site with minimal resistance; 2: orients to site with slight objection to manipulation; 3: withdraws from manipulation, may vocalize, may turn to guard area; 4: tries to escape from or prevent manipulation and may bite or show aggression. Joint pain scores for the appendicular joints of both hindlimbs were summed to create a “hindlimb” joint pain score, and similarly for the forelimbs. The pain scores of the forelimbs, hindlimbs, and axial skeleton regions were summed to create a total pain score. These values were used in the final analysis.

Muscle atrophy was evaluated by assessing individual muscles/muscle groupings (forelimb: supraspinatus, infraspinatus, triceps brachii, biceps brachii, antebrachium, and overall forequarters; hindlimb: gluteals, semimembranous/semitendinous/biceps femoris, crus and overall hindquarters) for both the left and right side. Each muscle grouping was evaluated for loss on a four-point scale (0–3): 0: none, normal, symmetric with opposite limb; 1: mild muscle loss felt on palpation; 2: moderate muscle loss felt and slightly visible; 3: severe muscle loss visible and can palpate underlying muscles. Additionally, an overall score for forequarters and hindquarters was recorded (using the same scale) and used in data analysis.

Each dog was categorized as predominately affected in the forelimbs or the hindlimbs based on veterinarian evaluation of appendicular pain. Spinal pain was an additional measure collected for completeness but *did not influence the forelimb or hindlimb classification*. As shown in Table 1, this classification is supported by significant differences in hindlimb pain and hindlimb atrophy scores among dogs with predominantly hindlimb impairments, and similarly, significant differences in forelimb pain and atrophy scores among dogs with predominantly forelimb impairments.

**Table 1:** Phenotypic characterization of study population.

Parameter		Hindlimb-predominant impairment (*n* = 74)	Forelimb-predominant impairment (*n* = 25)	Total population	*p*-value (hindlimb vs. forelimb dogs)
Age (years)	Mean (±SD)	9.31 (2.91)	9.70 (4.00)	9.41 (3.20)	0.6588
	Median (min, max)	9.55 (1.94, 14.94)	10.98 (0.50, 14.79)	10.01 (0.50, 14.94)	
Weight (kg)	Mean (±SD)	30.65 (7.25)	28.13 (12.35)	30.02 (8.82)	0.3412
	Median (min, max)	30.30 (16.50, 58.10)	26.00 (7.50, 59.00)	29.80 (7.50, 59.00)	
Sex	Female spayed	36 (48.64%)	13 (52%)	49 (49.49%)	
	Female intact	3 (4.05%)	1 (4%)	4 (4%)	
	Male castrated	34 (45.94%)	8 (32%)	42 (42.42%)	
	Male intact	1 (1.35%)	3 (12%)	4 (4%)	
BCS	Mean (±SD)	5.68 (1.11)	5.36 (0.86)	5.61 (1.06)	0.1317
	Median (min, max)	5.50 (3, 8)	5 (4, 7)	5 (3, 8)	
CBPI PSS	Mean (±SD)	4.53 (1.84)	4.33 (1.72)	4.48 (1.80)	0.6283
	Median (min, max)	4.63 (1.00, 9.00)	4.50 (1.25, 7.75)	4.5 (1.00, 9.00)	
CBPI PIS	Mean (±SD)	5.14 (2.09)	5.01 (2.33)	5.11 (2.15)	0.8017
	Median (min, max)	4.92 (1.50, 9.83)	4.83 (0.33, 9.33)	4.83 (0.33, 9.83)	
LOAD total	Mean (±SD)	27.19 (6.78)	25.96 (7.76)	26.88 (7.02)	0.4847
	Median (min, max)	26 (13, 47)	27 (10, 42)	26 (10, 47)	
Hindlimb pain score	Mean (±SD)	6.27 (2.54)	4.44 (2.62)	5.81 (2.67)	**0.0041**
	Median (min, max)	6 (2, 14)	4 (0, 10)	6 (0, 14)	
Forelimb pain score	Mean (±SD)	2.36 (2.85)	5.84 (3.85)	3.24 (3.46)	**0.0002**
	Median (min, max)	1 (0, 10)	5 (1,18)	2 (0, 18)	
Spinal pain score	Mean (±SD)	1.43 (2.36)	1.20 (1.44)	1.37 (2.16)	0.5613
	Median (min, max)	0 (0, 11)	0 (0, 4)	0 (0, 11)	
Total pain score (V1)	Mean (±SD)	10.39 (5.77)	11.32 (4.33)	10.63 (5.44)	0.4004
	Median (min, max)	9 (2, 32)	10 (5, 21)	9 (2, 32)	
Hind muscle atrophy	Mean (±SD)	2.18 (0.53)	1.56 (0.87)	2.02 (0.68)	0.0023
	Median (min, max)	2 (1, 3)	2 (0, 3)	2 (0, 3)	
Fore muscle atrophy	Mean (±SD)	0.97 (0.79)	1.60 (0.82)	1.13 (0.84)	**0.0018**
	Median (min, max)	1 (0, 3)	2 (0, 3)	1 (0, 3)	
Total muscle atrophy	Mean (±SD)	3.15 (1.09)	3.16 (1.43)	3.15 (1.18)	0.9714
	Median (min, max)	3 (1, 6)	3 (0, 6)	3 (0, 6)	

SD, standard deviation; kg, kilograms; BCS, Body Condition Score; LOAD, Liverpool in Osteoarthritis in Dogs questionnaire; CBPI, Canine Brief Pain Inventory questionnaire; PSS, Pain Severity Score; PIS, Pain Interference Score. Statistical difference between hindlimb and forelimb-predominant impairment groups was calculated using an unpaired, two-tailed *t*-test with an assumption of unequal variance (*α* = 0.05). Only hindlimb-and forelimb-specific parameters differed significantly between groups. Bold text indicates a *p*-value <0.05.

Sedated orthogonal radiographs were performed of all appendicular joints (mani, carpi, elbows, shoulders, pes, tarsi, stifles, hips) and the spine (cervical, thoracic, lumbar/lumbosacral). In general, dogs were sedated with butorphanol (0.2–0.3 mg/kg IV) and dexmedetomidine (0.003–0.005 mg/kg IV). Changes to sedation protocol were considered on an individual basis, such as in the case of cardiovascular disease. If radiographic signs of OA were noted in any joint deemed painful by the veterinarian during the orthopedic examination, OA-associated pain was considered present. Confirmatory radiographic findings included degenerative changes, osteophytes, subchondral sclerosis and bone remodeling.

#### 2.3.2 Owner assessments

Owner assessments of their dog’s level of pain and impairment were captured using Clinical Metrology Instruments (CMIs), also referred to as Client-Reported Outcome Measures (CROMs). These assessments included Client-Specific Outcome Measures (CSOM), Liverpool Osteoarthritis in Dogs (LOAD) and Canine Brief Pain Inventory (CBPI). All assessments were completed by the same owner on-site as previously described in the literature (31). Owners were instructed to complete each CMI based on observations of their dog over the previous 7 days. For each dog, the same owner was asked to complete CMIs for both visits to ensure consistency.

#### 2.3.3 CSOM

Owners were instructed to select three activities they observed to be challenging for their dog due to mobility changes, as previously reported (19, 31, 32). To be as specific as possible, a location and general time of day was provided for each activity (e.g., “jumping up on the living room couch in the morning in a smooth motion”). Owners were then asked to score how problematic it was for their dog to complete this activity, on average over the past 7 days, from the following categories: no problem, mildly problematic, moderately problematic, severely problematic, or impossible. To help owners appropriately categorize severity, an individual trained in CSOM assessments provided definitions and general descriptions for each level of impairment. Levels of difficulty were then transformed to numerical values from 0 to 4 (0: no problem; 1: mildly problematic; 2: moderately problematic; 3: severely problematic; 4: impossible). The scores were summed to confirm a minimum CSOM inclusion criterion of ≥5 across the 3 selected activities to enroll into the study population, thereby ensuring the study dogs had at least two activities that were, at a minimum, “moderately problematic.” CSOM assessments were performed at both the screening (Visit 1) and follow-up (Visit 2) to ensure consistent impairment within the study population. The CSOM was only used in determining eligibility for inclusion, not in data analysis.

#### 2.3.4 CBPI

The CBPI is a validated assessment of pain interference and severity in the dog (15, 33). It is comprised of two subcategories of questions—those relating to pain severity and those relating to how pain interferes with daily activity. This results in both a Pain Severity Score (CBPI PSS) and a Pain Interference Score (PIS). The PSS is the average of four items scored on an 11-point numerical scale (0 to 10, with increasing numbers indicating increased pain levels) which evaluate the dog’s pain at its worst, least, average, and as it is currently. The Pain Interference Score (CBPI PIS) is the average of six items, scored in the same manner as the CBPI PSS, which evaluate the role of pain in activity impairment. These six items ask the owner to assess impairment as it relates to general activity, enjoyment of life, and the ability to rise from lying down, walk, run, and climb up (e.g., stairs).

#### 2.3.5 LOAD

The LOAD instrument is a 13-item validated mobility assessment used to assess canine articular disorders including OA (16, 34). All items are reported on a five-point Likert-type scale and each item is scored between 0 and 4, where higher scores represent increased impairment. Item scores are summed to give an overall score for the instrument (LOAD total score). In general, the level of mobility impairment that can be assumed from the total LOAD score is as follows: mild (0–10), moderate (11–20), severe (21–30), extreme (31–52).

#### 2.3.6 Physical activity monitor

Dogs who met all inclusion criteria and scored ≥5 on CSOM evaluation were fitted with a collar-mounted accelerometer (Actigraph GT3X, Actigraph, Pensacola, Florida) to continuously record activity over a 10–14 day period in their home environment. Owners were asked to keep a diary of any unusual events that might affect the dog’s activity.

### 2.4 Follow-up visit (Visit 2)

#### 2.4.1 Veterinarian and owner assessments

Following the screening (Visit 1), there was a period of 10–14 days prior to the start of the study and randomization to therapeutic or placebo when baseline activity data were collected via accelerometry. Of note, regardless of the number of days that passed between Visit 1 and Visit 2, only accelerometry data from the 7 days prior to Visit 2 were included for analysis. Upon return to NCSU-CVM (Visit 2), the orthopedic examination was repeated by a clinical study veterinarian as described above. All CMI evaluations (CBPI, LOAD, CSOM) were repeated as previously described.

#### 2.4.2 Physical activity monitor

Data were downloaded upon return to NCSU-CVM (Visit 2) using proprietary software (ActiLife, Actigraph, Pensacola, Florida) on a computer. The collar-mounted GT3X sampled at a rate of 30 Hz, used a 12-bit analog to digital converter and stored data in a raw, non-filtered/accumulated format. Monitors were set to an epoch of 1-min (output set as “activity value” every minute). Output from the GT3X consists of values in the *x*, *y* and *z* directions as well as the average vector magnitude. The data were downloaded into spreadsheet files for further analysis (Excel, Microsoft, Redmond, Washington). Data from intervals where the accelerometer was not worn (according to owner diaries) were removed, as were any periods greater than 2 h where no motion was recorded (with the assumption the collar was not on the dog during these times). Periods where the owner stated the dog experienced a significant change in routine or activity (such as daycare or boarding) were also removed prior to analysis. In total, 2.2% of activity data were “missing” as a result of these processes. Regardless, each animal had a total of five 24-h periods (Monday through Friday) contributing to their “weekday” average and a total of two 24-h periods (Saturday and Sunday) contributing to their “weekend” average.

### 2.5 Data analysis

Demographic, examination, and owner assessment data were analyzed using statistical software (JMP 17, Cary, North Carolina). Descriptive statistics were reported using the mean, median, range, and standard deviation (SD). Comparison of variables were performed across dogs with predominately hindlimb impairment and those with predominately forelimb impairment using two-tailed *t*-tests with a significance threshold of 0.05. The experimental unit for this study was one dog.

#### 2.5.1 Functional linear modeling

Accelerometry data were analyzed using functional linear modeling (FLM) to evaluate the impact of various factors on 24-h activity patterns. Data from the most recent 7 days prior to Visit 2 were averaged to generate continuous, per-minute activity profiles for each dog, representing average activity over a 24-h period. To account for variations in activity due to owner interaction, data from weekends (Saturday and Sunday) were analyzed separately from weekdays (Monday–Friday) (35). One subject was excluded from the weekend analysis due to insufficient data collection.

To ensure the owner assessments, which reflect the dogs’ mobility over the previous 7 days, were aligned with the corresponding activity data, CMI data from Visit 2 were used. Visit 1 data were substituted in two cases where Visit 2 LOAD data were missing. All CBPI data were sourced from Visit 2. Orthopedic assessments, including forelimb and hindlimb pain scores, were taken from Visit 2 whenever possible; for seven subjects with missing data, scores from Visit 1 were used. Spinal pain assessments and thus total pain scores were recorded exclusively at Visit 1, in line with the original therapeutic study design.

A custom R package (“Actigraphy,” version 1.4.0, R Foundation for Statistical Computing, Vienna, Austria) was used to process and analyze the data (21). This package facilitated the matching of activity data to demographic, owner-reported, and veterinary examination covariates. The data were smoothed using a Fourier expansion, converting the raw minute-by-minute activity counts into functional representations. This smoothing process reduced variability and allowed for a continuous analysis of activity patterns over time.

FLM was applied to assess the effects of demographic parameters (age, body weight, and body condition score), owner-reported pain and mobility scores (CBPI and LOAD), and veterinary assessments (joint pain and muscle atrophy) on activity. Permutational *F*-tests (1,000 permutations per analysis) were used to identify statistically significant timepoints during the 24-h activity cycle. The results of the *F*-test are visualized as a red curve below the time-activity graph. The amplitude of this curve represents the magnitude of the *F* statistic (with higher *F* statistics corresponding to smaller *p*-values). Significance can be determined using either a pointwise or a maximum (global) critical value at the 0.05 threshold. The pointwise 0.05 critical value, represented by a dotted curved line, refers to the threshold of significance at individual time points across the activity curve. In contrast, the maximum 0.05 critical value, represented by a dashed straight line, provides a more conservative, global test of significance across the entire 24-h period. This global test accounts for the possibility of type 1 error due to multiple testing, ensuring that any significant differences observed are robust across the full time series. For this study, we considered a result to be significant when the *F* statistic exceeded the global (or “maximum”) critical value, which allows for a more conservative interpretation of the data.

#### 2.5.2 Additional correlation analysis

To further examine potential associations between variables, additional analyses to assess the relationship between body condition score (BCS) and clinical variables, including muscle atrophy, owner-reported pain scores (CBPI PIS, CBPI PSS, LOAD total score), and veterinary pain assessments (forelimb pain score, hindlimb pain score, spinal pain score) were performed. Pearson correlation analyses were used to evaluate the strength and direction of these relationships, and 95% confidence intervals were calculated. Similarly, we conducted sub-analyses to assess the effect of age on various clinical and owner-derived measures. Pearson correlations were performed for age versus BCS, CBPI PSS, CBPI PIS, LOAD total score, forelimb pain score, hindlimb pain score, and spinal pain score. For the latter three variables, a square root transformation was applied to meet the assumption of homoscedasticity required by the Pearson correlation. Additionally, for variables like muscle atrophy scores and LOAD total score, which stretched the assumption of continuity, a Kruskal–Wallis rank sum test followed by post-hoc pairwise tests (Wilcoxon rank-sum with a Benjamini–Hochberg correction for multiple comparisons) was conducted to evaluate differences across ordinal factor levels.

## 3 Results

### 3.1 Study population characteristics

One hundred and two client-owned animals were enrolled into the clinical study. Three dogs were excluded from the data analysis reported here: two were removed due to being fitted with an accelerometer unit that was not the GT3X model and the other for insufficient accelerometer data. Data from ninety-nine client-owned animals which had ≥7 consecutive days of activity data collected using the GT3X monitor were used. One dog was excluded from weekend analysis due to insufficient data but was included for weekday data. There was an almost equal female/male distribution (female spayed: 49; male neutered: 42; female intact: 4; male intact: 4). Thirty-one breeds of dog were represented; the three most common breeds were: Mixed breed (16, 16.2%); German Shepherd Dog (15, 15.2%) and Labrador Retriever (12, 12.1%). See Supplementary File 3 for further information on breed distribution. The mean age at study enrollment was 9.4 years (range 0.5–14.9, SD ± 3.2). Mean body weight was 30.0 kg (range 7.5–59.0, SD ± 8.8). Median BCS was 5 (range 3–8). No dogs with a BCS of 9/9 were enrolled due to study design. All descriptive statistics are reported in Table 1.

While data from Visit 2 was preferred for reflecting the previous 7 days of activity corresponding to the accelerometry data, imputation was required for a small subset of subjects where Visit 2 data was unavailable. Specifically, only 2 out of 99 patients (2.02%) required client-reported CMI data (LOAD) to be imputed from Visit 1, and 7 out of 99 patients (7.07%) required veterinary-reported pain scores to be similarly imputed. The decision to impute rather than omit these subjects was made to preserve the overall statistical power of the analysis. Imputation was performed under the assumption that the client’s perception of their dog’s pain and activity level would not have changed substantially between Visits 1 and 2 because no therapeutic intervention occurred between the visits. However, due to heightened owner awareness of their dog’s activities following the completion of the initial LOAD questionnaire at Visit 1, it would be reasonable to expect that the LOAD score at Visit 2 would be higher. To investigate our assumption, we performed a paired *t*-test for the LOAD scores between Visit 1 and Visit 2 for dogs that had data available for both days (*n* = 97). As expected, the LOAD score did have a statistically significant increase from Visit 1 to Visit 2, however, the difference in the mean was not clinically important (mean difference = 2.237, standard error of the mean = 0.3913) based on previously published work which defined a minimal clinically-important difference (MCID) of 4 for the LOAD assessment (34). Therefore, imputation was deemed a reasonable option. For veterinarian-determined pain scores, differences between Visit 1 and Visit 2 were not statistically different, which supported our decision to impute pain score data.

### 3.2 Owner assessments of severity of clinical signs

The CBPI pain severity score (CBPI PSS) was calculated by averaging the scores from questions 1–4 of the CBPI. Mean CBPI PSS was 4.5 (range 1.0–9.0, SD ± 1.8). CBPI pain interference score (CBPI PIS) was calculated by averaging CBPI questions 5–10. The mean CBPI PIS was 5.1 (range 0.3–9.8, SD ± 2.1). The mean CBPI “overall impression” score was 3.2 (range 1–5, SD ± 0.8), with higher numbers representing better quality of life. The mean total LOAD score was 26.9 (range 10–47, SD ± 7.0). CSOM results were only used to determine eligibility of dogs for study inclusion and were not included in further data analysis.

### 3.3 Veterinary assessments

Based on orthopedic assessment, 74/99 (74.7%) of dogs were noted to have predominantly hindlimb impairment and 25/99 (25.3%) had predominantly forelimb impairment. Mean forelimb total pain score was 3.2 (range 0–18, SD ± 3.5) and mean hindlimb total pain score was 5.8 (range 0–14, SD ± 2.7). Mean spinal pain score was 1.4 (range 0–11, SD ± 2.2). The mean total pain score was 10.6 (range 2–32, SD ± 5.4). The mean forelimb muscle atrophy score was 1.1 (range 0–3, SD ± 0.8) and mean hindlimb muscle atrophy score was 2.0 (range 0–3, SD ± 0.7). The mean total muscle atrophy score was 3.2 (range 0–6, SD ± 1.2). Full details are provided in Table 1.

### 3.4 Functional linear modeling

**Figure 2:**
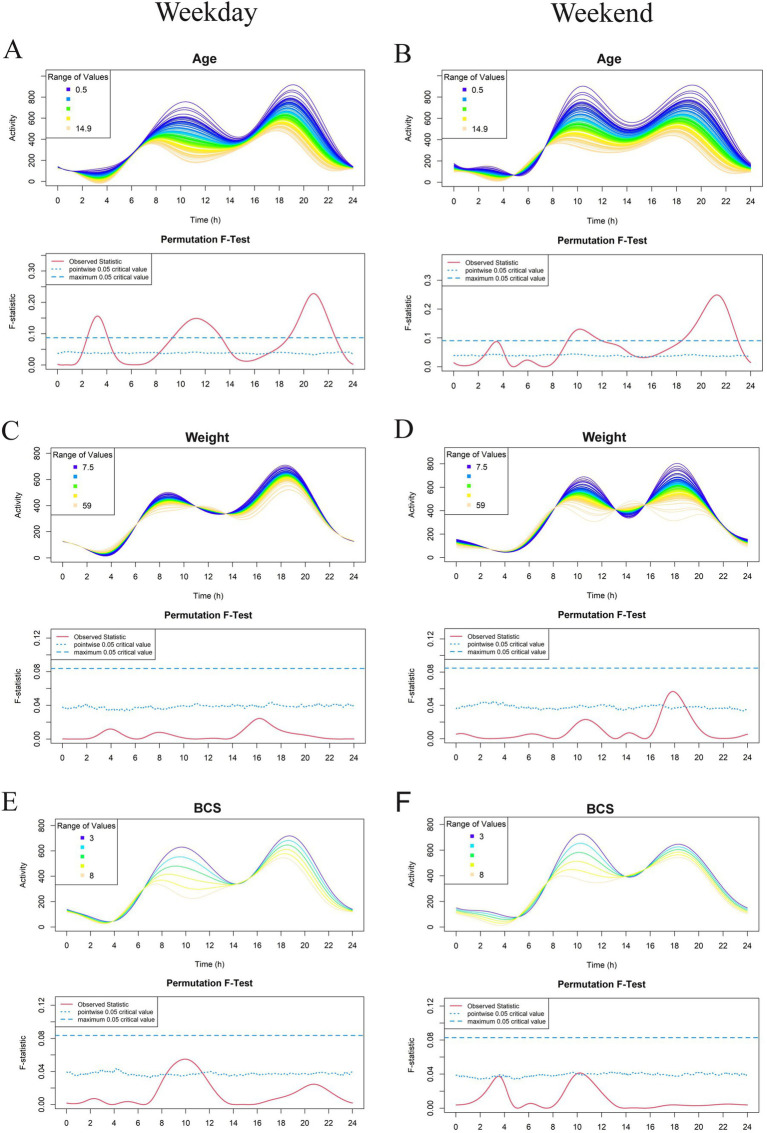
Effect of demographic parameters age **(A, B)**, weight **(C, D)**, and Body Condition Score (BCS) **(E, F)** on physical activity level in a study population of dogs with clinical evidence of osteoarthritis. The left-and right-hand columns correspond to weekday and weekend activity, respectively. For each parameter, the upper graph demonstrates activity over a 24-hour period and the lower Permutation *F*-test graph indicates the significance level of the differences. On this graph, the dotted (lower) and dashed (upper) blue lines indicate the pointwise and global (maximum) significance levels, respectively. When the solid red line is above the global 0.05 line, the differences between the values of the variable are considered significant. For weekday analyses, *n* = 99 and for weekday analyses, *n* = 98. All variables are treated as continuous for the *F* test.

**Figure 3:**
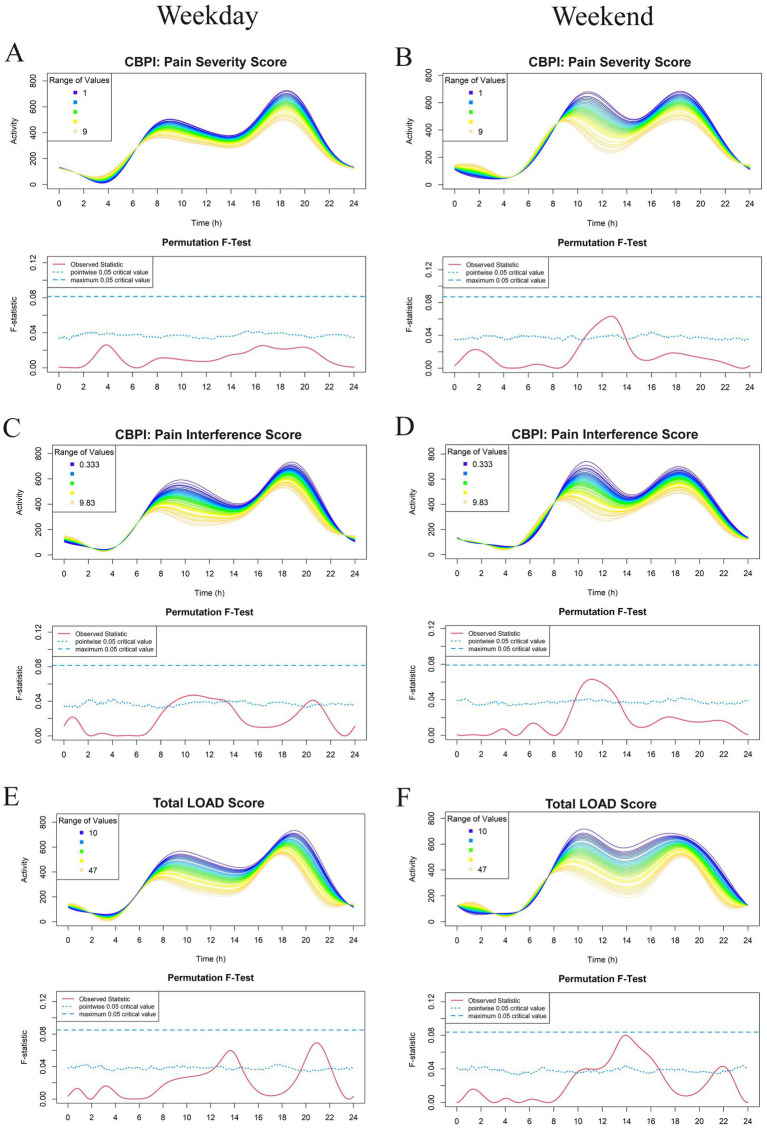
Effect of owner assessments: Canine Brief Pain Inventory (CBPI): Pain Severity Score (PSS) **(A,B)**, CBPI: Pain Interference Score (PIS) **(C,D)**, and Total Liverpool Osteoarthritis in Dogs (LOAD) **(E,F)** on physical activity levels in a study population of dogs with evidence of clinical osteoarthritis. The left-and right-hand columns correspond to weekday and weekend activity, respectively. For each parameter, the upper graph demonstrates activity over a 24-hour period and the lower Permutation *F*-test graph indicates the significance level of the differences. On this graph, the dotted (lower) and dashed (upper) blue lines indicate the pointwise and global (maximum) significance levels, respectively. When the solid red line is above the global 0.05 line, the differences between the values of the variable are considered significant. For weekday analyses, *n* = 99 and for weekday analyses, *n* = 98. All variables are treated as continuous for the *F* test.

**Figure 4:**
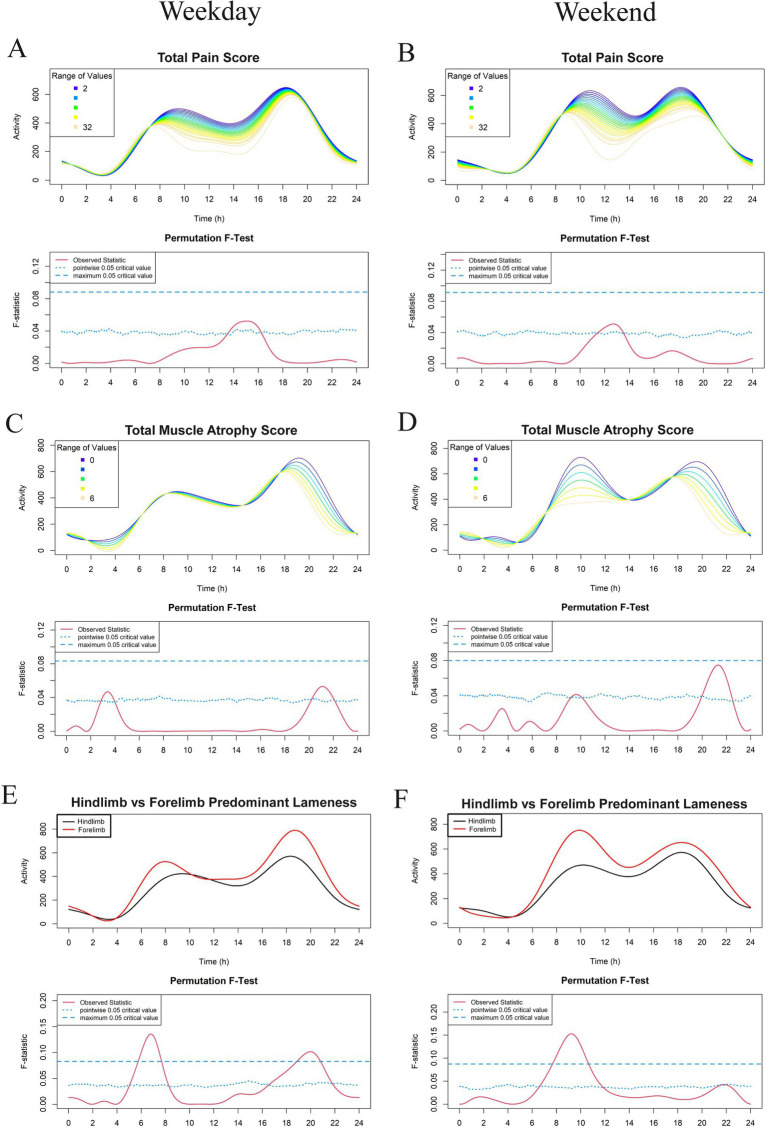
Effect of veterinary assessments total pain score **(A,B)**, total muscle atrophy scores **(C,D)**, and predominant limb lameness **(E,F)** on physical activity level in a study population of dogs with evidence of clinical osteoarthritis. The left-and right-hand columns correspond to weekday and weekend activity, respectively. For each parameter, the upper graph demonstrates activity over a 24-hour period and the lower Permutation *F*-test graph indicates the significance level of the differences. On this graph, the dotted (lower) and dashed (upper) blue lines indicate the pointwise and global (maximum) significance levels, respectively. When the solid red line is above the global 0.05 line, the differences between the values of the variable are considered significant. For weekday analyses, *n* = 99 and for weekday analyses, *n* = 98. Variables in panels **A–D** are treated as continuous for the *F* test, whereas those in panels **E,F** are treated as binary and are thus binned (weekday: *n* = 74 for hindlimb and *n* = 25 for forelimb; weekend: *n* = 73 for hindlimb and *n* = 25 for forelimb).

**Figure 5:**
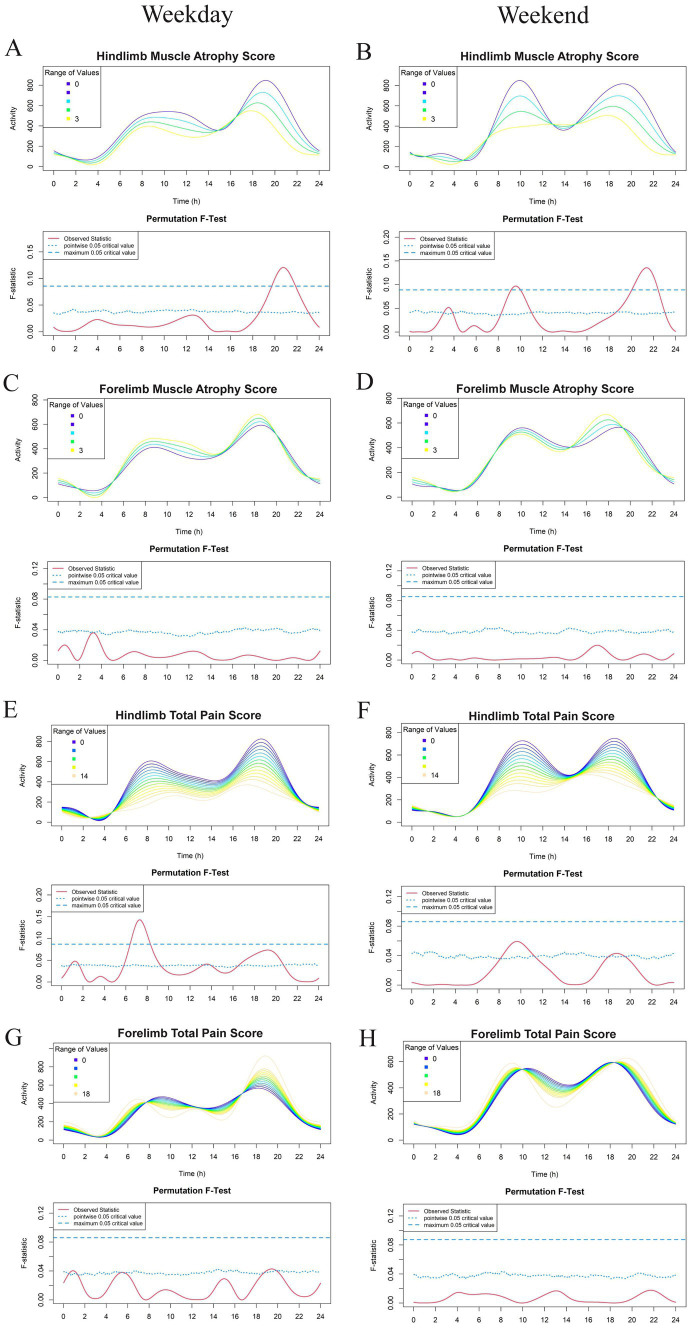
Effect of hind/forelimb muscle atrophy scores **(A–D)** and hind/forelimb total pain score **(E–H)** on physical activity levels in a study population of dogs with evidence of clinical osteoarthritis. The left-and right-hand columns correspond to weekday and weekend activity, respectively. For each parameter, the upper graph demonstrates activity over a 24-hour period and the lower Permutation *F*-test graph indicates the significance level of the differences. On this graph, the dotted (lower) and dashed (upper) blue lines indicate the pointwise and global (maximum) significance levels, respectively. When the solid red line is above the global 0.05 line, the differences between the values ofthe variable are considered significant. For weekday analyses, *n* = 99 and for weekday analyses, *n* = 98. All variables are treated as continuous for the *F* test.

The most recent 7 days of physical activity data, collected using the GT3X prior to Visit 2, were used for FLM analysis. Results are shown in Figures 2–5. Each subject contributed five 24-h periods to the “weekday” average curve and two 24-h periods to the “weekend” average curve.

For continuous variables (analyzed in Figures 2–5 except for Figures 4E,F), individual activity curves are binned in the graphs to enhance visual clarity. However, the *F* test was performed on all individual curves (*n* = 99 for weekdays and *n* = 98 for weekends). In the analysis shown in Figures 4E,F, subjects were categorized as predominantly forelimb-or hindlimb-impaired. Since this variable is binary, the forelimb group represents the average curve of 25 forelimb-predominant subjects, and the hindlimb group represents the average curve of 74 hindlimb-predominant subjects. In these graphs, the *F* test directly compares the smoothed curves of each group.

Looking overall at the patterns of activity, we see similar trends to those reported in previous studies (22–24). Weekday activity levels in dogs with OA fluctuated with established peaks of activity in the morning (between 6 and 9 am) and in the evenings (between 5 pm and 8 pm). A similar pattern occurred on the weekend, but with activity peaks occurring 1 to 3 h later than weekdays.

Within this cohort of dogs with OA pain who were deemed eligible for the therapeutic trial, age had the most pronounced effect on activity profiles. FLM modeling demonstrated a significant relationship between age and weekday activity in dogs with OA, with greater age reducing activity levels over the whole 24-h period. Older dogs were significantly less active during three periods: midnight to 4 am, 8 am to 2 pm, and after 8 pm. Similar effects were seen for weekend activity profiles (Figures 2A,B). Weight and BCS appeared to have a relationship with activity (weight in the early weekend evening and BCS during weekday mornings). However, neither of these variables were noted to have a significant effect (Figures 2C–F).

Overall, there appeared to be an inverse relationship with the majority of CMI scores (excluding CBPI: PSS, where no relationship was noted) and physical activity (Figure 3). Osteoarthritic dogs with higher CMI scores (greater owner-assessed pain and disability) had decreased activity during the late morning and early afternoon. Dogs with higher total LOAD scores were less active in the afternoon and evening on both the weekday and weekend; however, this relationship was not globally significant (Figures 3E,F). Because CMI scores are the average or aggregate of many individual questions, we evaluated whether any individual question scores appeared to be related to activity profiles in addition to the total summation scores. No individual questions in the CBPI reached the global level of significance (data not shown). The only individual CMI questions that had a significant effect on physical activity were LOAD questions (number 3 and 6) which ask about general activity level.

FLM analysis showed that OA dogs with higher total pain scores were less active during the early afternoon (Figures 4A,B) and dogs with higher total muscle atrophy scores were less active during the evening hours of 8 to 11 pm (Figures 4C,D). However, this relationship was not noted to be globally significant. Spinal pain was largely unassociated with activity levels apart from a short period just prior to midnight on weekdays in which dogs with higher spinal pain scores had decreased activity compared to those with lower spinal pain scores (Supplementary File 4).

Interestingly, dogs with predominantly hindlimb impairment were significantly less active than those with forelimb lameness during the morning hours of 6 am and 8 am and between 7 pm and 11 pm on weekdays. Weekends had a similar morning activity pattern in these dogs, with the peak shifted later by approximately 2 h (Figures 4E,F). Dogs with higher hindlimb muscle atrophy scores were less physically active during the weekday between 7 pm and 9 pm, as well as between 9 am and 11 am on the weekend (Figure 5). Dogs with higher total hindlimb pain scores were significantly less active on weekdays between 7 am and 9 am. There was no relationship between forelimb pain or muscle atrophy scores and physical activity.

### 3.5 Additional correlation analysis

Analysis of the relationship between BCS and clinical variables (Supplementary File 5) revealed a statistically significant positive correlation between higher BCS and hind limb muscle atrophy (r = 0.2124, 95% CI: 0.0097 to 0.3983, *p* = 0.02124). No significant correlations were observed between BCS and the CBPI PSS (*r* = 0.1625, 95% CI: −0.04201 to 0.3539, *p* = 0.1081), CBPI PIS (*r* = 0.1844, 95% CI: −0.01939 to 0.3735, *p* = 0.0676), LOAD total score (*r* = 0.1249, 95% CI: −0.08020 to 0.3199, *p* = 0.2179), forelimb pain score (*r* = 0.004194, 95% CI: −0.1991 to 0.2071, *p* = 0.9671), hindlimb pain score (*r* = 0.1394, 95% CI: −0.06553 to 0.3331, *p* = 0.1687), or spinal pain score (*r* = 0.05264, 95% CI: −0.1521 to 0.2530, *p* = 0.6048).

Regarding the relationship between BCS and age, no significant correlation was observed (*r* = −0.1002, 95% CI: −0.2919 to 0.09915, *p* = 0.3236), indicating that increased age does not necessarily result in higher BCS in this population. Age, however, was significantly correlated with several clinical and owner-reported measures, including LOAD total score (*r* = 0.4366, 95% CI: 0.2617 to 0.5837, *p* < 0.0001), CBPI PSS (*r* = 0.2222, 95% CI: 0.02598 to 0.4020, *p* = 0.0270), CBPI PIS (*r* = 0.3721, 95% CI: 0.1885 to 0.5305, *p* = 0.0001), forelimb pain score (*r* = 0.2427, 95% CI: 0.04758 to 0.4200, *p* = 0.0155), and spinal pain score (*r* = 0.2687, 95% CI: 0.07531 to 0.4427, *p* = 0.0072). No significant correlation was found between age and hindlimb pain score (*r* = 0.1432, 95% CI: −0.05577 to 0.3313, *p* = 0.1573).

Kruskal–Wallis rank sum tests (Supplementary File 6) confirmed significant associations between age and forelimb muscle atrophy scores (*p* = 0.001) and hindlimb muscle atrophy scores (*p* = 0.0042). The relationship between age and BCS also remained non-significant in non-parametric testing (*p* = 0.5984). Conversely, while the LOAD total score was significant in the Pearson correlation analysis, it lost significance in the Kruskal–Wallis test (*p* = 0.1014).

## 4 Discussion

Our study revealed significant relationships between certain demographic factors, owner assessments of pain and impairment, veterinary examination findings, and the physical activity profiles of dogs diagnosed with OA pain. However, our initial hypothesis—that increasing age, body weight, joint pain scores, and owner-reported pain and function scores (CBPI and LOAD) would significantly reduce activity profiles—was not uniformly supported. Age emerged as a key factor, with older dogs showing lower levels of physical activity at peak periods throughout the day. Dogs with predominately hindlimb impairment, greater hindlimb muscle atrophy, and higher hindlimb pain scores also had significantly reduced activity levels. In contrast, while higher CMI scores, total pain scores, and total muscle atrophy scores tended to be associated with decreased activity at peak periods, these associations did not reach statistical significance. These findings are consistent with the broader literature, which suggests a decline in activity levels as OA pain progresses in dogs (6, 22) and as age increases (24). Interestingly, no significant relationship with activity was found for forelimb OA, weight, or BCS.

One of the key methodological advancements of our study is the application of functional linear modeling (FLM) to analyze high-frequency longitudinal activity data. Unlike traditional methods that often rely on summary statistics or bucketed activity data, FLM allows for a more nuanced analysis of temporal patterns in activity, revealing specific time points where factors like age, weight, and pain severity exert significant effects. Gruen et al. (22) highlighted the limitations of traditional linear mixed-effects models in capturing these details, advocating for FLM as a superior method to identify differences in activity patterns, particularly during critical periods such as nighttime and early morning. Our findings support this approach, demonstrating that FLM is particularly effective in capturing the impact of age and pain on daily activity patterns in dogs with OA.

### 4.1 General assessment of activity profiles

The global patterns of activity seen within this cohort of osteoarthritic dogs is consistent with work previously reported. Canine diurnal activity is well established, both within laboratory populations, working and free-living dogs, and client-owned animals (4, 36, 37). Weekend physical activity patterns are influenced by the human-animal interaction, and the rightward shift of weekend physical activity seen in our work has also been observed in FLM modeling of feline activity (25, 35).

Interestingly, the 24-h activity profile of humans in western cultures mirrors that of dogs on the weekend, with one large peak occurring from around 9 am to 9 pm. This likely reflects the fact that activity patterns in dogs are highly influenced by owner activity, and most owners work Monday–Friday but remain home on the weekends. This highlights the importance of separating weekdays from weekends to best capture dog-directed changes in activity patterns rather than owner-directed ones.

In the human literature, accelerometry is often used to assess mobility in patients with osteoarthritis. However, unlike in animal models, this is usually performed in the context of monitoring response to recommended increases in physical activity (38–41). One study does report inherent decreases in physical activity in people with early osteoarthritis, prior to being recommended exercise interventions (42). These studies primarily use either a bucketed approach or linear mixed models rather than FLM. Instead, in human literature, FLM is most frequently applied in the context of circadian rhythms and sleep disturbance (43, 44) and represents a difference in the goal of accelerometry in dogs versus people. In dogs, activity monitoring and FLM analysis are critical methods for bolstering objectivity in pain research, since we cannot ask our canine companions to fill out a questionnaire, and owner questionnaires that are available are subjective.

### 4.2 Effect of age on activity profiles

The significant reduction in physical activity observed in older dogs in our study is consistent with findings from previous research, including Mondino et al. (24) and Woods et al. (23), who reported that older dogs show lower activity levels during specific periods, namely the late afternoon and evening. Our FLM analysis revealed that older osteoarthritic dogs were less active during the midnight to 4 am, 8 am to 2 pm, and after 8 pm periods on both weekdays and weekends. These findings suggest that the impact of age on OA-related activity reduction is pervasive throughout the day, rather than limited to specific times. Our study also supported previous observations of a leftward shift in the bimodal distribution of activity, where the two peaks occur earlier in the day for older dogs (24). The age-related decline in activity could be attributed to factors such as an overall reduced motivation to engage in physical activity or cognitive dysfunction, both of which are well-documented trends in geriatric canine populations (22, 24).

### 4.3 Impact of CMI scores

There are few studies in the literature that directly measure the relationship between Clinical Metrology Instrument (CMI) scores and accelerometry in dogs. However, clinical trials demonstrate that as CMI scores improve in response to therapy, so do activity levels. For instance, Foster et al. (45) quantified changes in CMIs and activity levels in dogs undergoing treatment for idiopathic immune-mediated polyarthropathy and observed a concurrent decrease in CBPI scores and increase in daily activity levels. Similarly, Lascelles et al. (46) reported increased daytime activity in dogs treated with an anti-nerve growth factor antibody, which also corresponded with improvements in owner-reported pain and function scores.

Our research demonstrated a similar inverse relationship between CMI scores and physical activity levels, particularly during late morning and early afternoon periods. However, these did not reach the global level of significance. Nevertheless, a few individual CMI questions that focused on characterizing activity levels (LOAD questions 3 and 6) did show significant associations with activity patterns (data not shown). Specifically, higher owner-reported activity levels corresponded with higher accelerometer-measured activity, which supports the validity of these questions.

In our cohort of dogs with OA pain, the average CBPI PIS was 5.1, and the CBPI PSS was 4.5. These results are closely aligned with those reported in other therapeutic studies for OA, such as Brown et al. (33, 47), who reported median baseline PIS and PSS values ranging from 3.92–4.33 and 3.50–4.25, respectively (depending on the randomized treatment group), as well as Muller et al. (31) and Lascelles et al. (48), who found average baseline PIS and PSS values of 4.2 and 4.5, respectively. Similarly, Muller et al. (31) and Lascelles et al. (48) found the LOAD scores in their study to average around 20.3, which is comparable to the average LOAD score of 26.9 observed in our study. These consistent findings across multiple studies suggest that our cohort of dogs with OA pain experience a similar degree of pain and mobility impairment to those reported at baseline in other therapeutic trials. Given this consistency we anticipate future FLM analyses of accelerometric physical activity data serving as the baseline for a larger interventional study, with FLM analyses of the randomized interventional study groups in the same cohort of dogs with OA.

### 4.4 Additional correlation analysis

Additional correlational sub-analyses provide further context to the relationship between body condition score (BCS), age, and clinical metrics in dogs with osteoarthritis. Higher BCS was weakly but significantly correlated with hind limb muscle atrophy, likely reflecting the biomechanical challenges faced by obese dogs in maintaining hind limb muscle mass. However, no significant correlations were observed between BCS and owner-reported pain severity, pain interference, or activity-related metrics. These findings suggest that while BCS may influence certain clinical aspects like muscle atrophy, it does not directly impact pain perception or mobility as assessed by owners or activity monitors. Importantly, the lack of a significant correlation between BCS and age further supports that obesity alone is not a primary driver of reduced activity patterns in this cohort. In contrast, age was significantly associated with LOAD scores, owner-reported pain metrics (CBPI PSS and PIS), and some clinical pain scores (forelimb and spinal). These findings highlight age as a major driver of clinical outcomes, including worsening muscle atrophy, higher pain scores, and reduced mobility. Interestingly, no significant correlation was observed between age and hindlimb pain scores, which may suggest compensatory mechanisms or different load-bearing patterns as dogs age.

### 4.5 Significance of orthopedic and muscle atrophy findings

Our study found that dogs with predominant hindlimb impairment exhibited significantly lower activity levels during the morning hours (6 am to 8 am on weekdays, with a delayed peak on weekends). Hindlimb pain had the biggest effect on physical activity during the morning hours while hindlimb atrophy had noted effects during evening hours and morning hours on the weekend. The forelimbs carry approximately 60% of total mechanical load and are primarily responsible for braking, while the hindlimbs are mainly responsible for propulsion (49). As such, the hindlimbs are important for initiating and maintaining motion. This may explain why activity levels were affected by hindlimb OA but not forelimb OA. Marshall et al. (50) demonstrated the critical role of mechanical load on the hindlimbs in dogs with forelimb lameness, with the hindlimbs taking on more breaking force when dogs had forelimb pain. This is interesting in light of our study, as we found in subanalyses (data not shown) that hindlimb pain and muscle atrophy maintained significant relationships with activity even when only including dogs with predominate forelimb lameness. The association between hindlimb muscle atrophy and decreased physical activity underscores the importance of maintaining muscle mass in managing OA-related disability in both forelimb and hindlimb predominate patients. These results suggest that targeted rehabilitation strategies aimed at preserving or enhancing muscle mass in the hindlimbs could be beneficial in improving mobility and quality of life in dogs with OA.

### 4.6 Implications for therapeutic approaches

Measuring ground reaction forces using force plate or pressure sensitive walkway analysis is often considered the ideal standard for gait evaluation; however, these instruments are expensive and require specific expertise, training and rigorous methodology for appropriate data collection. Additionally, the animal is only evaluated at a singular point in time outside their normal home environment, which impacts the translational nature of these results (47, 51). Lastly, these techniques are best applied in cases of single, or predominately single, limb dysfunction since multiple limb dysfunction often gets cancelled out. In contrast, accelerometry can be deployed in the home environment, has demonstrated acceptable correlation with movement (18), provides the opportunity for evaluation over periods of time, and will capture global influences on activity (e.g., multi-joint OA pain). As previously discussed by Lascelles et al. (12), continued development of and understanding of objective instruments that measure direct change while in the home environment over a prolonged interval are critical for the advancement of diagnosis, study and management of chronic pain in companion animals.

While previous research has established the link between OA pain and overall reduced physical activity in dogs, our study provides novel insights by employing functional linear modeling (FLM) to assess the impact of various factors on activity profiles. Previous studies have used accelerometry to monitor activity in dogs with OA, but they have often relied on summary statistics, which can overlook the temporal nuances in activity patterns. Our application of FLM allows for a more detailed temporal analysis, capturing subtle changes in activity that might be missed by conventional methods.

The findings from our study have implications for the evaluation of OA therapeutics. By demonstrating the utility of FLM in capturing detailed activity profiles, we provide a framework for integrating this approach into clinical trials. This could enhance the sensitivity of outcome measures, by targeting assessments of activity on specific time periods, or controlling for specific covariates in analysis. Understanding the influence of various factors on outcome assessments is critical to optimizing their utilization.

Recent reviews have highlighted the potential for the use of companion animal canines as a high-fidelity model for translational chronic pain research (12, 28, 52, 53). Advancing our understanding of how to measure the impact of OA pain in this model is critical. The lack of success in developing new analgesics for human OA pain, combined with the high prevalence and significant impact of OA pain in people, highlights the need for more relevant models (3, 54–56). Gaining a greater understanding of the influences on activity in canine naturally occurring OA pain is important because activity is clearly impacted in humans with OA, and mobility is an important feature of quality of life in people (55).

### 4.7 Study limitations and future directions

While our study provides valuable insights into the impact of demographic and clinical factors on activity profiles in dogs with OA, limitations must be acknowledged. To start, this study looked at factors influencing activity patterns *within a cohort of dogs with OA pain*. While we believe our results are generalizable to other populations of dogs with OA pain, having a cohort of dogs with no OA-associated pain to compare would provide additional insights into the specific effects of OA pain on activity levels. Additionally, the exclusion criteria, particularly the exclusion of dogs with extreme body condition scores (BCS 9/9), may have introduced a selection bias that could affect the applicability of the results to the broader canine population. Future studies could consider including a more diverse population to validate and extend our findings.

Another limitation of our study is the reliance on accelerometry data, which, while providing objective measures of physical activity, may be subject to inaccuracies due to variations in owner compliance and owner-controlled influences. Although owners were asked to keep diaries of events that might unusually affect activity (e.g., collar removed, bathing, car rides), owners may not have remembered to capture all such events. Additionally, owner lifestyle (e.g., household size, retirement, working from home) was not considered in this study. Further research could explore the potential for integrating additional data sources, such as owner-reported digital diaries or video monitoring and variables related to owner demographics, to enhance the understanding of OA pain on dog activity profiles. The use of permutation *F*-tests in our FLM analysis, while robust, may have limitations in terms of statistical power, as it is unable to account for multiple variables.

To build on the insights gained from our study, we recommend several avenues for future research. Longitudinal studies that extend beyond the 7-14-day monitoring period used in our study could provide a more comprehensive understanding of how OA-pain-related activity patterns evolve over time. Moreover, conducting multi-cohort comparisons across different breeds, age groups and body conditions could help identify specific subpopulations of dogs that may be more or less responsive to certain therapeutic interventions. Finally, investigating the role of owner behavior and interaction patterns in shaping activity levels could provide valuable information for developing more effective and personalized OA management strategies.

## 5 Conclusion

These findings underscore the importance of integrating advanced analytical techniques like FLM into clinical research to enhance our understanding of outcome measures, which in turn will enhance our understanding of the clinical utility of therapeutics. As the field of veterinary medicine continues to evolve, such approaches will be crucial in developing more effective strategies for managing OA-related pain and improving the quality of life for affected dogs.

## Data Availability

The raw data supporting the conclusions of this article will be made available by the authors, without undue reservation.

## Supplementary material

Supplemental File 1 Screening visit diagnostic data.

Supplemental File 2 DCFdata.

Supplemental File 3 Breeds data.

Supplemental File 4 Spinal pain data.

Supplemental File 5 Pearson correlations data.

Supplemental File 6 Kruskal-wallis data.
