# Supplementary material for: Factors influencing, and associated with, physical activity patterns in dogs with osteoarthritis-associated pain
Source: Front Vet Sci. 2025 Mar 19;12:1503009. doi: 10.3389/fvets.2025.1503009 (PMC11963776; doi:10.3389/fvets.2025.1503009)
Supplement: Supplemental File 2 — DCFdata. [file Data_Sheet_2.pdf]

NC State Translational Research in Pain (TRiP) Canine Musculoskeletal Examination
(Gait, Pain, Muscle Mass, Neuro)

Translational Research in Pain Program

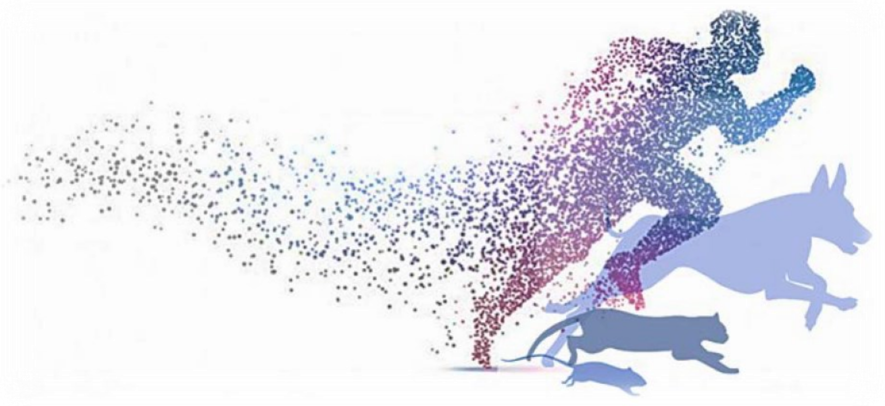

NC STATE

©2024 North Carolina State University, Translational Research in Pain (TRiP)

Gait, posture and limb use assessment

Place Patient Sticker Here

Case Number: ______________________________

Date: _____________________

Individual Limb Use Assessment:

		R Forelimb	L Forelimb	R Hindlimb	L Hindlimb
Walk	Lameness (0-4)				
Trot	Lameness (0-4)				
Posture	Standing (0-4)				

Key:
Lameness:
0: none, weight bearing observed on all strides; normal gait
1: Slight, subtle lameness, weight may be slightly shifted to one side on some strides
2: Mild, obvious lameness with moderate weight bearing on all strides, may toe touch occasionally
3: Moderate, consistent minimal weight bearing on most strides
4: Severe non-weight bearing during gait
Posture:
0: Normal stance;
1: Slight off-loading (favors limb but foot on floor; difficult to see)
2: Mild offloading (favors lib but foot on floor; obvious)
3: Moderate off loading
4: Severe off-loading - not using limb at all

Global Gait and Posture Evaluation:

Parameter	Assessment
Walk	
Trot	
Posture	

Key:
Gait/Movement:
0: Normal ; 1: Slightly abnormal ; 2: Mildly abnormal; 3: Moderately abnormal; 4: Severely abnormal

Posture:
0: Normal ; 1: Slightly abnormal ; 2: Mildly abnormal; 3: Moderately abnormal; 4: Severely abnormal

NC STATE ©2024 North Carolina State University, Translational Research in Pain (TRiP)

Muscle atrophy assessment

Place Patient Sticker Here

Case Number: ______________________________

Date: ______________________

Muscles	R Forelimb	L Forelimb	Muscles	R Hindlimb	L Hindlimb
Supraspinatus			Gluteals		
Infraspinatus			Semimembranous/ Semitendinous/ Biceps femoris		
Triceps Brachii					
Biceps Brachii			Quadriceps Femoris		
Antebrachium			Crus		
Overall Forequarters†			Overall hindquarters†		

Muscle Evaluation*:

* Muscles Atrophy
0: None, normal, symmetric with opposite limb
1: mild muscle loss felt on palpation
2: moderate muscle loss is felt and slightly visible
3: severe, muscle loss is visible can palpate one or underlying muscles

† This score is not the addition of all other scores, but a separate 'overall' score for the forelimbs/hindlimbs

NC STATE ©2024 North Carolina State University, Translational Research in Pain (TRiP)

Joint Evaluation Scoring SystEm (JESSE$_{canine}$)

R Forelimb	Pain (0-4)	Crepitus (0-2)	Effusion (0-2)	Thickening (0-2)	ROM (0-2)
Manus					
Carpus					
Elbow					
Shoulder					
R Hindlimb	Pain (0-4)	Crepitus (0-2)	Effusion (0-2)	Thickening (0-2)	ROM (0-2)
Pes					
Hock					
Stifle					
Hip					
L Forelimb	Pain (0-4)	Crepitus (0-2)	Effusion (0-2)	Thickening (0-2)	ROM (0-2)
Manus					
Carpus					
Elbow					
Shoulder					
L Hindlimb	Pain (0-4)	Crepitus (0-2)	Effusion (0-2)	Thickening (0-2)	ROM (0-2)
Pes					
Hock					
Stifle					
Hip					

Spinal Column	Pain (0-4)
Cervical	
Thoracic	
T-L	
Lumbar	
L-S	

The descriptors on the following scoring systems are considered a guide and are especially useful for studies and situations where different individuals are making assessments on the same patient over time. Ideally, a single trained individual makes all the assessments on every patient in a given study.

NC STATE ©2024 North Carolina State University, Translational Research in Pain (TRiP)

Pain scale based on passive flexion, extension and manipulation

0: Does not notice manipulation
1: Orients to site on manipulation, does not resist or only mild resistance (mild)
2: Orients to site, slight objection to manipulation (moderate)
3: Withdraws from manipulation, may vocalize, may turn to guard area (significant)
4: Tried to escape from manipulation, or prevent manipulation, may bite or show aggression on manipulation (severe)

Crepitus, Effusion, Thickening and Range of Motion (ROM) based on passive flexion, extension and manipulation:

Crepitus:
0: none
1: mild, occasional crepitus
2: moderate, crepitus felt always
3: severe, can feel and hear crepitus

Effusion:
0: none
1: mild, small fluid pocket felt only on careful palpation
2: moderate, prominent/obvious on palpation
3: severe, may see visible fluid pocket

Thickening:
0: none, can feel all anatomic structures easily
1: mild, less defined anatomic structures
2: moderate, can still discern the detail of anatomic structures of the joint
3: severe, can no longer feel anatomic structures of the joint

Range of motion:
0: normal
1: mild-moderate decrease
2: severely decreased

NC STATE ©2024 North Carolina State University, Translational Research in Pain (TRiP)

Basic neurological examination

Place Patient Sticker Here

Case Number: ______________________________

Date: _____________________

Gait (description)<br>normal, ataxic, paretic, lame				
Are there any neurologic abnormalities that affect gait?				
	Forelimb		Hindlimb	
Score each of the clinical signs below on a 0-4 scale *	Left	Right	Left	Right
Hopping				
Patella reflex				
Withdrawal reflex				
Hemi-walking				
* 0 – absent; 1 – reduced; 2 – normal; 3 – increased; 4 - clonus				

Panniculus (0 to 4)*		Level of start:
Spinal pain [yes/no]		If yes, where:

Spinal Pain	Cervical	Thoracic	T-L	Lumbar	L-S
(0-4) †					

†Pain based on manipulation – 5 point scale

0: Does not notice manipulation; 1: Orients to site on manipulation, does not resist or mild resistance (mild); 2: Orients to site, slight objection to manipulation (moderate); 3: Withdraws from manipulation, may vocalize, may turn to guard area (significant); 4: Tries to escape from manipulation, or prevent manipulation, may bite or show aggression on manipulation (severe)

NC STATE ©2024 North Carolina State University, Translational Research in Pain (TRiP)
