# Supplementary material for: Factors influencing, and associated with, physical activity patterns in dogs with osteoarthritis-associated pain
Source: Front Vet Sci. 2025 Mar 19;12:1503009. doi: 10.3389/fvets.2025.1503009 (PMC11963776; doi:10.3389/fvets.2025.1503009)
Supplement: Supplemental File 3 — Breeds data. [file Data_Sheet_3.pdf]

frontiers

Supplementary Material

Supplemental File 3. Breed distribution of study population

Breed	Total	Total %
**American Staffordshire Terrier**	5	5.05%
**Australian Cattle Dog**	1	1.01%
**Australian Cattle Dog mix**	1	1.01%
**Australian Shepherd**	5	5.05%
**Bassett Hound**	2	2.02%
**Border Collie**	3	3.03%
**Border Collie mix**	2	2.02%
**Boxer**	2	2.02%
**Boxer mix**	1	1.01%
**Chow Chow**	2	2.02%
**Collie**	1	1.01%
**Coonhound mix**	1	1.01%
**Foxhound**	1	1.01%
**German Shepherd Dog**	15	15.15%
**German Shepherd mix**	2	2.02%
**GSD/Golden Retriever mix**	1	1.01%
**Giant Schnauzer**	1	1.01%
**Golden Retriever**	6	6.06%
**Great Pyrenees**	2	2.02%
**Labrador Retriever**	12	12.12%
**Labrador mix**	6	6.06%
**Mixed Breed Dog**	16	16.16%
**Newfoundland**	1	1.01%
**Pembroke Welsh Corgi**	1	1.01%
**Pitbull mix**	2	2.02%
**Pug**	1	1.01%
**Retriever**	1	1.01%

**Saluki**	1	1.01%
**Siberian Husky**	2	2.02%
**Terrier mix**	1	1.01%
**Wirehaired Pointing Griffon**	1	1.01%
**Total**	99	100.00%
