# Supplementary material for: Factors influencing, and associated with, physical activity patterns in dogs with osteoarthritis-associated pain
Source: Front Vet Sci. 2025 Mar 19;12:1503009. doi: 10.3389/fvets.2025.1503009 (PMC11963776; doi:10.3389/fvets.2025.1503009)
Supplement: Supplemental File 4 — Spinal pain data. [file Data_Sheet_4.pdf]

Supplementary Material

<u>WEEKDAY</u> <u>WEEKEND</u>

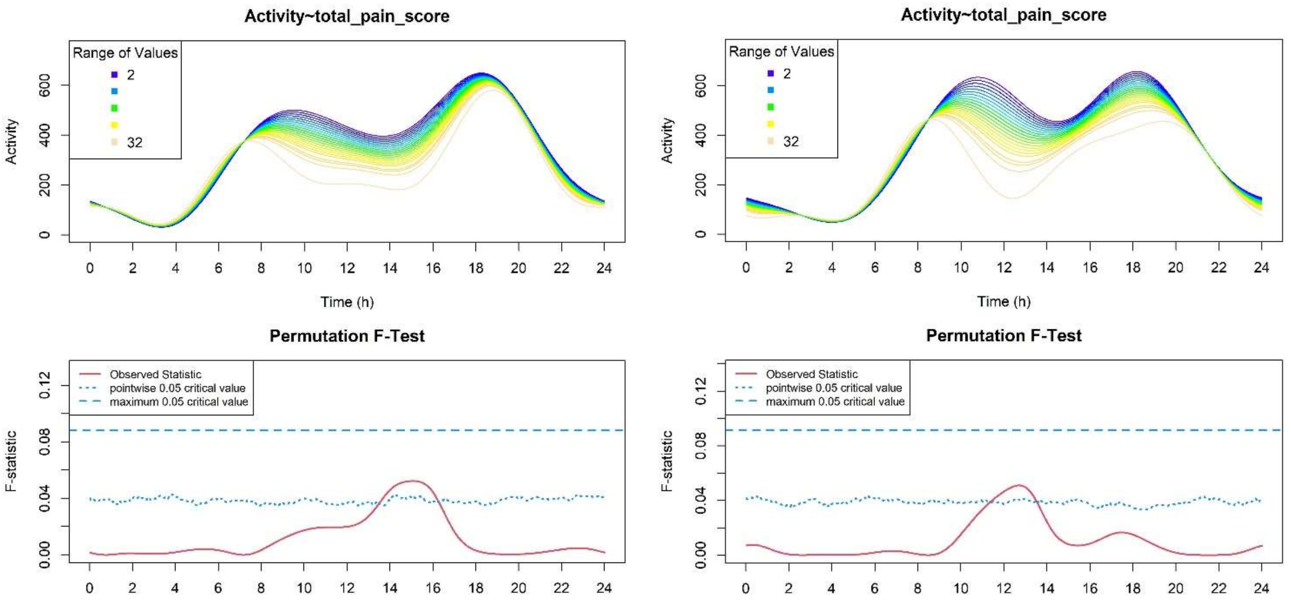

Supplemental File 4. Effect of "total spinal pain score" (as determined by veterinary examination) on physical activity levels. For each set of graphs (Weekday, left; and Weekend, right), the upper graph demonstrates activity over a 24-hour period and the lower Permutation F-test graph indicates the significance level of the differences. The dotted (lower) and dashed (upper) blue lines indicate the pointwise and global (maximum) significance levels, respectively. When the solid red line is above the global 0.05 line, the differences between the values of the variable are considered significant. For weekday analyses, n = 99 and for weekday 848 analyses, n = 98. All variables are treated as continuous for the F test.
