# Supplementary material for: Factors influencing, and associated with, physical activity patterns in dogs with osteoarthritis-associated pain
Source: Front Vet Sci. 2025 Mar 19;12:1503009. doi: 10.3389/fvets.2025.1503009 (PMC11963776; doi:10.3389/fvets.2025.1503009)
Supplement: Supplemental File 5 — Pearson correlations data. [file Data_Sheet_5.pdf]

Supplementary Material

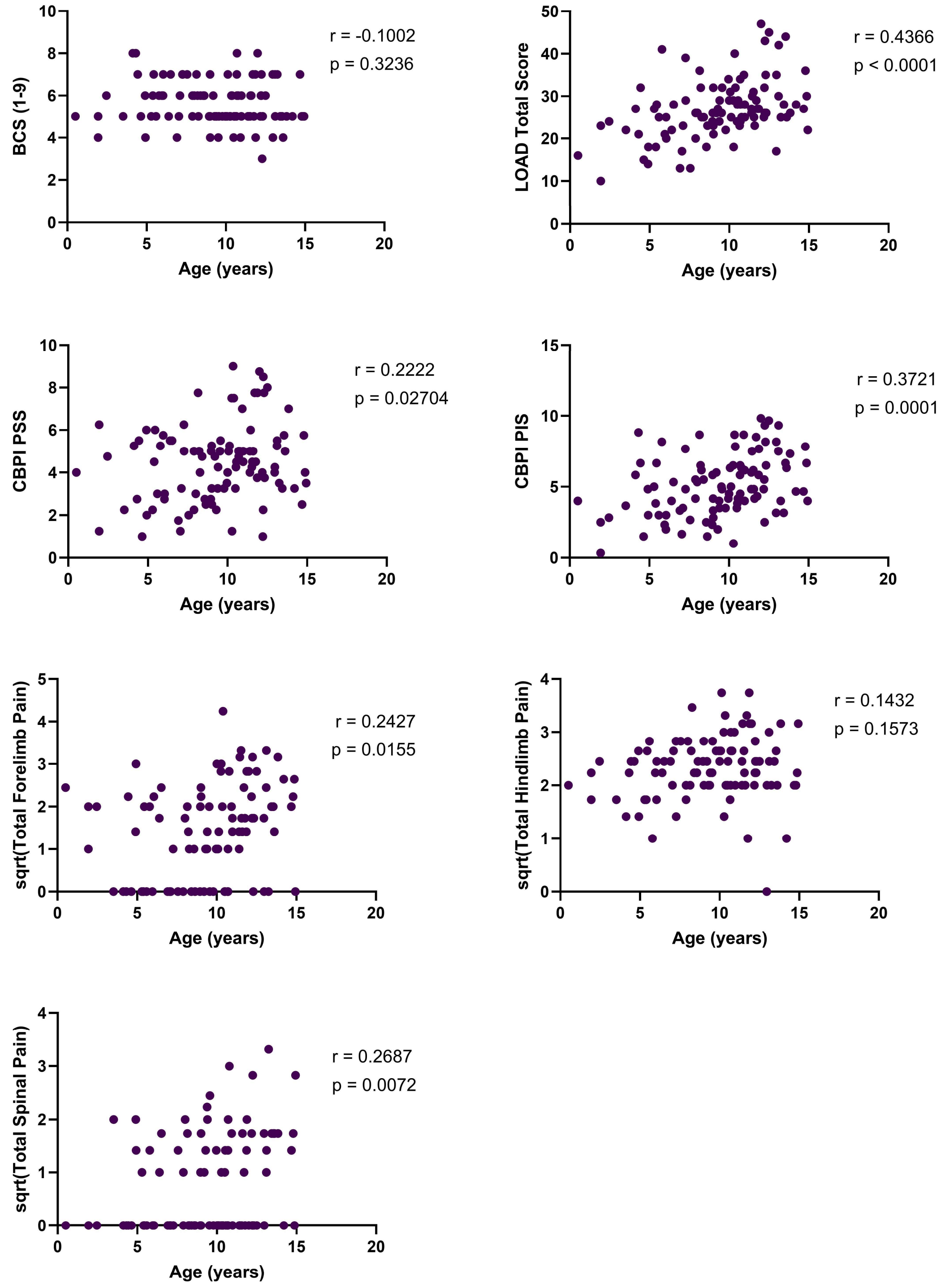

Supplement File 5. Correlations between body condition score (BCS, 1-9) and select clinical and owner-determined variables. *Statistical test: Pearson correlation, alpha = 0.05.*
