# Supplementary material for: Factors influencing, and associated with, physical activity patterns in dogs with osteoarthritis-associated pain
Source: Front Vet Sci. 2025 Mar 19;12:1503009. doi: 10.3389/fvets.2025.1503009 (PMC11963776; doi:10.3389/fvets.2025.1503009)
Supplement: Supplemental File 6 — Kruskal-wallis data. [file Data_Sheet_6.pdf]

Supplementary Material

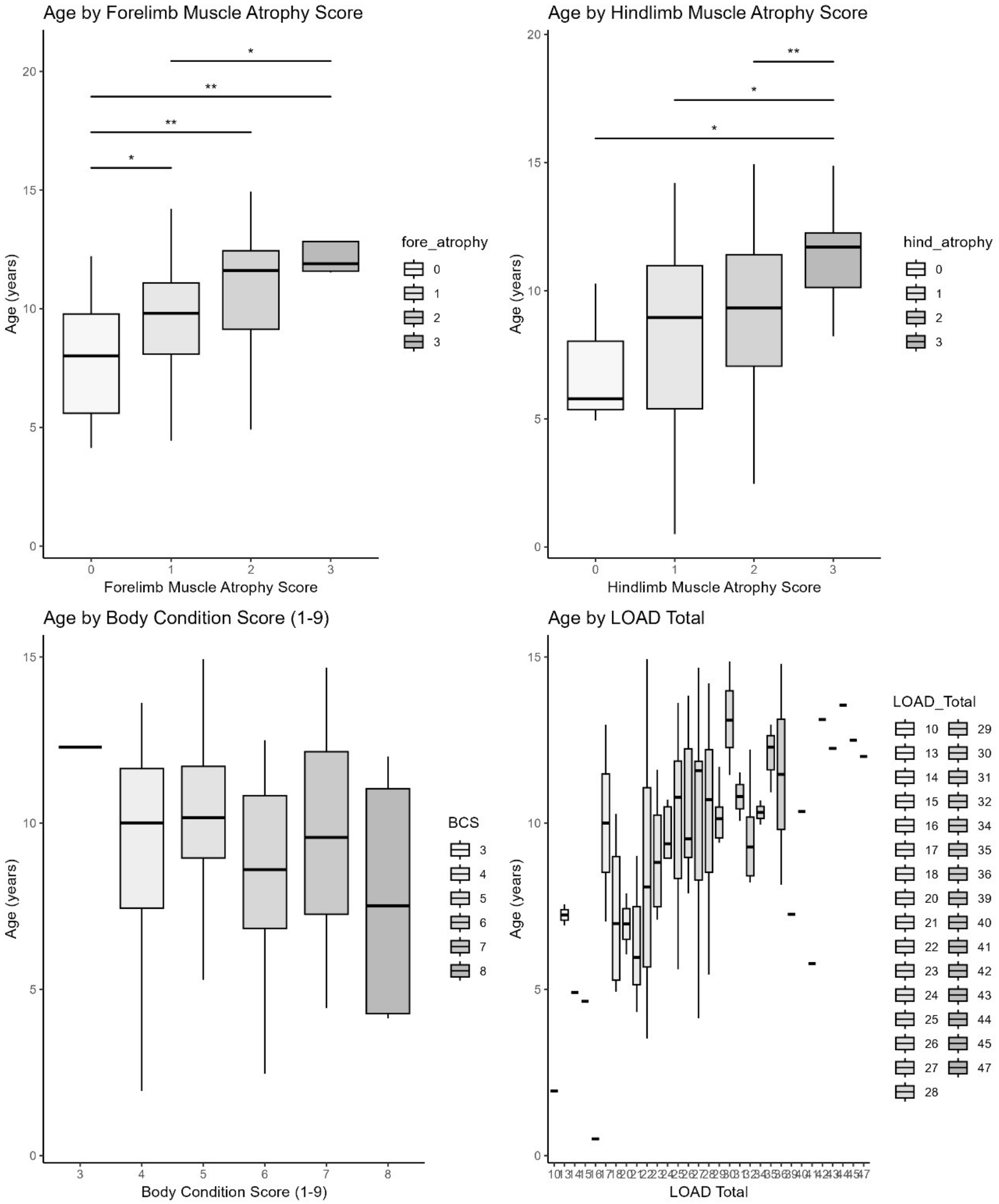

Supplemental File 6. Relationship between factor levels of select variables (forelimb muscle atrophy score, hindlimb muscle atrophy score, body condition score, LOAD total score) and age as a continuous variable. *Key: * = p<0.05, ** = p<0.01, *** = p<0.0001. Statistical test: Kruskal-Wallis rank sum test for general significance followed by Wilcoxon rank-sum pairwise comparisons with Benjamini-Hochberg correction for multiple comparisons), alpha = 0.05.*
